# Data quality in population-based cancer registration: an assessment of the Merseyside and Cheshire Cancer Registry.

**DOI:** 10.1038/bjc.1997.443

**Published:** 1997

**Authors:** D. J. Seddon, E. M. Williams

**Affiliations:** Public Health Medicine, North West Regional Health Authority, University of Liverpool, UK.

## Abstract

Merseyside and Cheshire Cancer Registry (MCCR) data quality was assessed by applying literature-based measures to 27,942 cases diagnosed in 1990 and 1991. Registrations after death (n = 8535) were also audited (n = 917) to estimate death certificate only (DCO) case accuracy and the proportion of registrations notified by death certificate (DC). Ascertainment appeared to be high from the registration/mortality ratio for lung [1.01:1] and to be low from capture-recapture estimates (59.4%), varying significantly with site from oesophagus [92.2% (95% CI 88.5-95.9)] to breast [47.5 (95% CI 41.8-53.2)]. The estimated DC-dependent proportion was 20% (5601 out of 27 942) with successful traceback in 3533 out of 5601 (63.1%) cases. DCO flagging (2497 out of 27,942, 8.9%) overestimated true DCO cases (2068 out of 27,942, 7.4%). The proportion of cases of unknown primary site was low (1.5%), varying significantly with age [0-4.2%, (95% CI 2.5-5.9)] and district [0.8% (95% CI 0.3-1.3) to 2.2% (95% CI 1.8-2.6)]. The median diagnosis to registration interval appeared to be good (10 weeks), varying significantly with site (P < 0.0001), age (P < 0.0001) and district (P < 0.0001). The proportion with a verified diagnosis was 77.3%, varying significantly with site [lung 55.2% (95% CI 53.7-56.7) to cervix 96.9% (95% CI 96.3-97.5)], age [45.2% (95% CI 40.9-49.5) to 97.5% (95% CI 96.4-98.6)] and district [71.8% (95% CI 69.9-73.8) to 82.5% (95% CI 80.7-84.3)]. The DCO percentages varied similarly by site [non-melanoma skin 0.4% (95% CI 0.2-0.6) to lung 22.6% CI (95% 19.9-25.3)], age [0.7(95% CI 0.1-1.4) to 23.0 (95% CI 19.4-26.6)] and district [6.9% (95% CI 5.7-8.1) to 13.9% (95% CI 12.9-15.0)]. MCCR data quality varied with age, site and district - inviting action - and apparently compares favourably with elsewhere, although deficiencies in published data hampered definitive assessment. Putting quality assurance into practice identified shortcomings in the scope, definition and application of existing measures, and absent standards impeded interpretation. Cancer registry quality assurance should henceforward be within an explicit framework of agreed and standardized measures.


British Journal of Cancer (1997) 76(5), 667–674
© 1997 Cancer Research Campaign

### Data quality in population-based cancer registration: an assessment of the Merseyside and Cheshire Cancer Registry

**DJ Seddon**[1] **and EMI Williams**[2]

[1]Public Health Medicine, North West Regional Health Authority; [2]Merseyside and Cheshire Cancer Registry, Muspratt Building, University of Liverpool, PO Box 147, Liverpool L69 3BX, UK

**Summary** Merseyside and Cheshire Cancer Registry (MCCR) data quality was assessed by applying literature-based measures to 27 942 cases diagnosed in 1990 and 1991. Registrations after death ($n$ = 8535) were also audited ($n$ = 917) to estimate death certificate only (DCO) case accuracy and the proportion of registrations notified by death certificate (DC). Ascertainment appeared to be high from the registration/mortality ratio for lung [1.01:1] and to be low from capture–recapture estimates (59.4%), varying significantly with site from oesophagus [92.2% (95% CI 88.5–95.9)] to breast [47.5 (95%CI 41.8–53.2)]. The estimated DC-dependent proportion was 20% (5601 out of 27 942) with successful traceback in 3533 out of 5601 (63.1%) cases. DCO flagging (2497 out of 27 942, 8.9%) overestimated true DCO cases (2068 out of 27 942, 7.4%). The proportion of cases of unknown primary site was low (1.5%), varying significantly with age [0–4.2%, (95% CI 2.5–5.9)] and district [0.8% (95% CI 0.3–1.3) to 2.2% (95% CI 1.8–2.6)]. The median diagnosis to registration interval appeared to be good (10 weeks), varying significantly with site ($P$ < 0.0001), age ($P$ < 0.0001) and district ($P$ < 0.0001). The proportion with a verified diagnosis was 77.3%, varying significantly with site [lung 55.2% (95% CI 53.7–56.7) to cervix 96.9% (95% CI 96.3–97.5)], age [45.2% (95% CI 40.9–49.5) to 97.5% (95% CI 96.4–98.6)] and district [71.8% (95% CI 69.9–73.8) to 82.5% (95% CI 80.7–84.3)]. The DCO percentages varied similarly by site [non-melanoma skin 0.4% (95% CI 0.2–0.6) to lung 22.6% CI (95% 19.9–25.3)], age [0.7(95% CI 0.1–1.4) to 23.0 (95% CI 19.4–26.6)] and district [6.9% (95% CI 5.7–8.1) to 13.9% (95% CI 12.9–15.0)]. MCCR data quality varied with age, site and district – inviting action – and apparently compares favourably with elsewhere, although deficiencies in published data hampered definitive assessment. Putting quality assurance into practice identified shortcomings in the scope, definition and application of existing measures, and absent standards impeded interpretation. Cancer registry quality assurance should henceforward be within an explicit framework of agreed and standardized measures.

**Keywords:** cancer registry; quality control; standards

Population-based cancer registration aims to register every patient diagnosed with cancer within a defined geographical area (Freedman, 1978) to inform health policy makers, service purchasers and providers, and researchers. In England and Wales, 12 regional registries form the largest population-based cancer register in the world (Swerdlow, 1986). Started in 1944, Merseyside and Cheshire Cancer Registry (MCCR – previously Mersey Regional Cancer Registry) registers about 13 000 new cancers annually from a population of 2.4 million (Youngson et al, 1992), receiving initial information from pathology laboratories, death certificates (DCs) mentioning cancer and hospital information systems. Further information about the individual, the tumour and its clinical management, and outcome, is obtained from hospitals, general practitioners, nursing homes, private hospitals, hospices and the Office of National Statistics (ONS – previously the Office of Population Censuses and Surveys, OPCS). MCCR routinely supplies an agreed Cancer Minimum Data Set (NHS Management Executive, 1992) to ONS and collects additional data, especially about treatment, for local use.

*Received 27 June 1996*
*Revised 12 February 1997*
*Accepted 27 February 1997*

*Correspondence to:* EMI Williams

The importance of quality assurance is emphasized internationally in 'Cancer Incidence in Five Continents' (Parkin and Muir, 1992) and, in the UK, by its inclusion in regional corporate contracts for cancer registration (NHS Management Executive, 1996).

Four attributes of registry data quality are described. *Ascertainment* is 'the degree to which reportable incident cases ... are actually recorded in the registry' (Robles et al, 1988). It is variously termed 'accuracy', 'completeness', 'completeness of registration' and 'completeness of ascertainment' by authors. The use of 'ascertainment' alone avoids confusion with completeness of detail and is consistent with the English meaning (Collins Concise English Dictionary, 1982). *Completeness* (of detail) describes the extent to which all appropriate data items have been recorded (Skeet, 1991). *Timeliness* describes the currency of registry data. *Validity* is 'the proportion of cases recorded with a given characteristic (sex, age, diagnosis) which truly has this attribute' (Parkin and Muir, 1992).

MCCR ascertainment has previously been reported as 88.7% for childhood cancers compared with 91.8% in England and Wales (Hawkins and Swerdlow, 1992). Incidence to mortality ratios for lung cancer were 1.12 and 1.11 for men and women, respectively, in 1989, compared with equivalent ratios for England and Wales of 1.06 and 1.07 (OPCS, 1994). These measures, although limited, imply ascertainment worse than the national average in MCCR. Similarly, MCCR ranks seventh out of nine UK registries submitting data internationally for the overall proportion histologically verified: 66% men and 71% women compared with equivalent figures from the Oxford Regional Registry of 76% and 80% (Parkin and Muir, 1992), implying suboptimal validity. The dearth of information about the quality of MCCR data, and the implication from the small amount published that improvement is needed, prompted the aims of this study, i.e. to identify data quality measures from the literature, apply them to MCCR data and make recommendations for quality assurance extendable to other registries.

### MATERIALS AND METHODS

Published papers, reference volumes, OPCS publications and reports from UK and overseas registries were searched to identify measures of cancer registry data quality. Measures of ascertainment, completeness, timeliness and validity were selected according to their reproducibility (adequately described with few assumptions, clear definitions and simple calculations), practicability (relevant information collected by MCCR) and potential comparability with data from other registries.

Data items, e.g. age at diagnosis, cancer site coded to three digits in ICD-O [International Classification of Diseases for Oncology (Version 1)] and district of residence at diagnosis, were selected according to their association with previously identified measures on all registered cases diagnosed in 1990 and 1991 to form the study database. The measures were then applied to the study database and analysed by district of residence, age at diagnosis and cancer site.

For measures requiring information about notification source, which was not computerized in MCCR, the paper records of cases registered after death were audited separately to determine those cases that were *uniquely* dependent on the DC for notification and the accuracy of death certificate only (DCO) cases identification. For cases registered from a DC, by local convention, date of death is also entered on to the computer as date of diagnosis until more information becomes available, thereby flagging DCO cases. Four random samples of registrations after death were selected, according to the presence or absence of the DCO flag and the length of the death to registration interval. Sample sizes were calculated for a population survey with a single parameter having an estimated frequency of 50%. Sample paper records were searched manually for independent source documents, forming an audit database of key identifying variables and information about notification source. Representativeness was confirmed by comparing the frequencies of five variables (sex, age group, site, year of diagnosis and district of residence) between each sample and the study database. Death certificate-dependent (DCD) registrations were defined as those containing a death certificate as the *only* notifying paper document. Records containing notifications from other sources (e.g. pathology), even if *first* notified by DC, were regarded as independently notified. DCDs were reclassified as DC-plus registrations when registry enquiry (traceback) yielded additional information, and DCO registrations when traceback was unsuccessful.

When possible, results are presented with confidence limits, calculated using standard formulae (Gardner and Altman, 1992). Statistical significance was assessed using the Kruskal–Wallis test for analysis of variance and Spearman's rank correlation coefficient.

### RESULTS

Ten measures were identified from the literature (Table 1), all lacking explicit standards. The study database contained 27 942 primary cancers diagnosed in 1990 and 1991, of which 8535

**Table 1** Measures of quality assurance

| Quality assurance area | Measure | Source | Comment |
|---|---|---|---|
| Ascertainment | Registration – mortality ratio | Balarajan and Scott (1983) | |
| | | Swerdlow (1986) | See appendix for worked example |
| | Capture–recapture | Robles et al (1988) | |
| | Death certificate – dependent proportion (DCD%) | Freedman (1978)<br>Goldberg et al (1980)<br>Skeet (1991) | Cases uniquely notified by death certificate |
| | Death certificate – notified proportion (DCN%) | Benn et al (1982)<br>Parkin et al (1994) | Cases first notified by death certificate |
| Completeness | Primary site–unknown proportion | Parkin and Muir (1992) | |
| | Proportion of cases with missing information | Parkin and Muir (1992) | As missing date of birth and sex |
| Timeliness | Diagnosis to registration interval | Thames Cancer Registry (1992) | As median and 75th percentile |
| Validity | Proportion of cases with a verified diagnosis | Parkin and Muir (1992) | As sum of histopathological proof, cytological proof and other 'special tests'; e.g. blood films and special imaging |
| | Death certificate-only proportion (DCO%)[a] | Parkin and Muir (1992)<br>Black et al (1993) | Approximated by the DCO flagged proportion |
| | | Parkin and Muir (1992) | |
| | Consistency checks | Skeet (1991) | e.g. Between sex and site, dates of diagnosis and registration |

[a]DCO% is also a measure of completeness.

© Cancer Research Campaign 1997

**Table 2** Cases registered after death: weighted estimates in the study population ($n$ = 8535) based on audited records ($n$ = 917)

| Category | DCO flagged | | | | No DCO flag | | | | Totals | |
|---|---|---|---|---|---|---|---|---|---|---|
| | Death registration interval | | | | Death registration interval | | | | | |
| | < 6 weeks | | ≥ 6 weeks | | < 6 weeks | | ≥ 6 weeks | | | |
| | *n* | (%) | *n* | (%) | *n* | (%) | *n* | (%) | *n* | (%) |
| Population | 2333 | (100) | 164 | (100) | 5530 | (100) | 508 | (100) | 8535 | (100) |
| (Sample selected) | (320) | | (123) | | (357) | | (217) | | (1017) | |
| (Sample audited) | (281) | | (105) | | (335) | | (196) | | (917) | |
| Death certificate independent | 257 | (11.0) | 36 | (22.0) | 2361 | (42.7) | 280 | (55.1) | 2934 | (34.4) |
| Death certificate plus | 125 | (5.4) | 11 | (6.7) | 3169 | (57.3) | 228 | (44.9) | 3533 | (41.4) |
| Death certificate only | 1951 | (83.6) | 117 | (71.3) | – | – | – | – | 2068 | (24.2) |
| Death certificate dependent | 2076 | (89.0) | 128 | (78.0) | 3169 | (57.3) | 228 | (44.9) | 5601 | (65.6) |

**Table 3** Registration to mortality (R/M) ratios by district in Mersey NHS region 1990 and 1991 (both sexes) for cancers of the stomach (ICD[a] 151), trachea, bronchus and lung (ICD 162)

| District | Site | | | |
|---|---|---|---|---|
| | Registered cases | | R/M ratio | |
| | Stomach | Lung | Stomach | Lung |
| 1 | 53 | 219 | 1.39 | 1.01 |
| 2 | 95 | 325 | 1.46 | 1.03 |
| 3 | 298 | 1223 | 1.32 | 1.19 |
| 4 | 177 | 622 | 1.30 | 1.14 |
| 5 | 176 | 684 | 1.26 | 1.05 |
| 6 | 76 | 250 | 1.33 | 1.04 |
| 7 | 117 | 321 | 1.30 | 1.01 |
| 8 | 56 | 227 | 1.33 | 1.06 |
| 9 | 83 | 260 | 1.15 | 1.17 |
| 10 | 86 | 267 | 1.26 | 1.19 |
| Mersey region | 1217 | 4398 | 1.30 | 1.11 |

Source: Mersey Regional Cancer Registry data 1990–91 and OPCS series DH5 'Mortality in England and Wales by area, 1990 and 1991'. [a]ICD, International Classification of Diseases.

(32.2%) were registered after death. The audit samples comprised 1017 out of 8535 (11.9%) cases, of which 917 out of 1017 (90.2%) were successfully traced, forming the audit database. The distribution of attributes (age, sex, site of cancer, district of residence, year of diagnosis) in the audit samples was comparable with those in the four parent populations. Table 2 shows the weighted estimates for the study database derived from the audit.

### Ascertainment

Overall, Mersey registration to mortality (R/M) ratios were 1.30:1 and 1.11:1 for stomach (ICD-O 151) and lung cancer (ICD-O 162) respectively. District ratios for lung and stomach cancers within Mersey region (Table 3), although variable, exceeded unity; ratios for stomach cancer were appropriately and consistently higher than those for lung, reflecting better survival.

Table 4 shows considerable variation between sites for capture–recapture estimates. The 'all sources' estimate of ascertainment (59.4% overall) was greater than the 'two source' estimate (52.5% overall), because cases ascertained from other sources (e.g. hospital information systems) were included. Ascertainment appeared low for sites like female breast in which overlap between sources was small, i.e. when survival is longer and hence there are fewer DC notifications. When overlap was greater, e.g. for lung, ascertainment appeared to be higher. For sites with effectively a single source (e.g. testis – pathology only), the method failed as there was no 'recapture'.

From the audit, the DCD% (cases *uniquely* notified by DC) was 65.6% (5601 out of 8535) of registrations after death and 20% (5601 out of 27 942) overall, comprising 24.2% (2068) DCO cases and 41.4% (3533) DC-plus cases (Table 2). The DC-notified proportion (DCN%) (cases *first* notified by DC) was an estimated 96.7% (8255 out of 8535) of cases registered after death and 29.5% (8255 out of 27 942) overall, comprising 5601 DCD cases, 293 DC-independent cases (DCO flagged and thus triggered by a DC) and 2361 unflagged cases registered within 6 weeks of death (likely to have been triggered by a DC). An estimated 2934 (34.4%) registrations after death were DC independent.

### Completeness

The proportion of cases with an unknown primary site varied significantly among districts (Table 5) and with age, being low initially in children and young adults but increasing thereafter (Table 6). Date of birth and sex were missing in 13 and two records (< 0.01%) respectively.

### Timeliness

The median diagnosis to registration interval for Mersey region was 10 weeks. An exceptional interval of 24 weeks in district 7 compared with a range from 8 to 12 weeks in other districts (Kruskall–Wallis $P < 0.0001$) (Table 5). The interval decreased with increasing age (Spearman rank $P < 0.0001$) (Table 6) and varied significantly with site from 8 weeks (oesophagus) to 16 weeks (brain) (Table 7). The median and 75th percentile values varied similarly by age, site and district.

### Validity

Among districts, the proportion with a verified diagnosis varied significantly from 71.8% to 82.5%, with a regional average of 77.3% (Table 5). Verification varied significantly with age,

© Cancer Research Campaign 1997

Table 4 Estimated ascertainment by selected site using capture–recapture methods, Mersey Regional Cancer Registry 1990–91

| Site (ICD-O[a]) | Number of registered cases | Percentage ascertainment (95% confidence limits) | | | |
|---|---|---|---|---|---|
| | | Two source[b] | | All sources[c] | |
| Oesophagus (150) | 623 | 89.1 | (85.5–92.6) | 92.2 | (88.5–95.9) |
| Stomach (151) | 1217 | 84.2 | (81.2–87.2) | 86.3 | (83.2–89.4) |
| Colon (153) | 1790 | 62.3 | (58.6–66.1) | 65.5 | (61.6–69.5) |
| Rectum (154) | 1070 | 70.5 | (65.9–75.0) | 75.9 | (71.0–80.8) |
| Larynx (161) | 228 | 74.3 | (62.5–86.1) | 91.1 | (76.6–100) |
| Trachea, bronchus and lung (162) | 4398 | 81.2 | (79.1–83.3) | 85.7 | (83.4–87.9) |
| Haematopoietic tissue (169) | 921 | 50.9 | (43.7–58.1) | 86.2 | (74.0–98.4) |
| Non-melanomatous skin (173) | 4627 | 62.1 | (56.0–68.3) | 70.8 | (63.8–77.8) |
| Female breast (174) | 2749 | 36.4 | (32.0–40.8) | 47.5 | (41.8–53.2) |
| Uterine cervix (180) | 2783 | 46.8 | (38.6–55.1) | 58.7 | (48.4–69.1) |
| Uterine body (182) | 355 | 55.6 | (44.2–66.9) | 69.7 | (55.5–84.0) |
| Ovary (183) | 460 | 70.5 | (63.3–77.7) | 82.9 | (74.5–91.4) |
| Prostate (185) | 1122 | 62.7 | (57.7–67.8) | 69.6 | (64.0–75.2) |
| Testis (186) | 117 | 81.1 | (30.7–100) | 100 | (52.1–100) |
| Bladder (188) | 1160 | 64.9 | (59.8–70.1) | 73.1 | (67.3–79.0) |
| Brain (191) | 399 | 70.9 | (62.8–79.0) | 77.5 | (68.6–86.4) |
| Unknown primary (199) | 406 | 69.6 | (37.0–100) | 71.9 | (38.2–100) |
| All sites (140–208, excluding 172) | 27 942 | 52.5 | (51.4–53.5) | 59.4 | (58.2–60.6) |

[a]International Classification of Diseases for Oncology – Version 1. [b]Two-source method: observed cases notified from pathology and death certificate notifications. [c]All-source method: observed cases as for two-source method plus clinically notified cases. In both methods, expected cases are calculated using the capture–recapture method (Appendix).

Table 5 Quality indicators in Mersey Regional Cancer Registry 1990–91: district of residence

| District | Cases | Verification of diagnosis[a] | | DCO flag[b] | | Primary site unknown | | Diagnosis to registration interval (weeks) | | |
|---|---|---|---|---|---|---|---|---|---|---|
| | | % | (95% CL) | % | (95% CL) | % | (95% CL) | Median[c] | (95% CL) | 75th percentile |
| 1 | 1746 | 82.5 | (80.7–84.3) | 6.9 | (5.7–8.1) | 1.0 | (0.5–1.5) | 8 | (7–8) | 14 |
| 2 | 1973 | 76.8 | (74.9–78.7) | 10.9 | (9.5–12.3) | 1.9 | (1.3–2.5) | 12 | (11–12) | 20 |
| 3 | 6592 | 80.0 | (79.0–81.0) | 8.3 | (7.7–9.0) | 1.2 | (0.9–1.5) | 10 | (10–10) | 17 |
| 4 | 3961 | 78.3 | (77.0–79.6) | 9.2 | (8.3–10.1) | 1.3 | (1.0–1.7) | 10 | (9–10) | 15 |
| 5 | 4326 | 73.2 | (71.9–74.5) | 13.9 | (12.9–15.0) | 2.2 | (1.8–2.6) | 9 | (8–9) | 15 |
| 6 | 2010 | 80.1 | (78.4–81.9) | 7.2 | (6.0–8.3) | 1.0 | (0.6–1.5) | 8 | (7–8) | 14 |
| 7 | 2052 | 71.8 | (69.9–73.8) | 6.2 | (5.2–7.3) | 2.0 | (1.4–2.6) | 24 | (22–25) | 56 |
| 8 | 1463 | 78.0 | (75.9–80.1) | 7.0 | (5.7–8.4) | 0.8 | (0.3–1.3) | 10 | (9–10) | 16 |
| 9 | 1972 | 77.6 | (75.8–79.4) | 7.2 | (6.0–8.3) | 1.1 | (0.6–1.6) | 9 | (8–9) | 18 |
| 10 | 1847 | 72.6 | (70.6–74.6) | 6.9 | (5.8–8.1) | 1.4 | (0.9–1.9) | 10 | (9–10) | 17 |
| All districts | 27 942 | 77.3 | (76.8–77.8) | 8.9 | (8.6–9.3) | 1.5 | (1.4–1.6) | 10 | (10–10) | 17 |

[a]Verification includes pathological and cytological proof as well as proof by 'special test'. [b]The Registry DCO flag overestimated 'true' DCO registration – see text. [c]Kruskal–Wallis $\chi^2$ = 1340 (9 d.f.), $P < 0.0001$. 95% CL, 95% confidence limits.

increasing to a plateau between ages 25 and 34 years and thereafter decreasing (Table 6). Table 7 shows significant variation in verification by site, from 55.2% (lung) to 96.9% (cervix).

There were an estimated 2497 out of 8535 (29.3%) DCO-flagged cases and 2068 out of 8535 (24.2%) true DCO cases based on the audited sample, giving overall DCO proportions of 8.9% (2497 out of 27 942) and 7.4% (2068 out of 27 942), respectively, 8 months after the end of the study period (Table 2). The DCO flag had a positive predictive value estimate of 83% (2068 out of 2497), thereby acting as a reasonable proxy for true DCO cases, and varied with death to registration interval from 71% (117 out of 164) under 6 weeks to 84% (1951 out of 2333) at or over 6 weeks. The 429 DCO flag errors (1.5% overall) arose through the misclassification of 293 (68%) DC-independent cases and 136 (32%) DC-plus cases. There were significant differences between districts for DCO-flagged cases, with a twofold difference between the extreme values (Table 5). The DCO-flagged proportion varied significantly with age, initially decreasing in children and young adults, thereafter increasing (Table 6), and varied significantly with site, from less than 2.5% in larynx, cervix and skin to 22.6% for haematopoietic malignancies and 56.4% when the primary site is unknown (Table 7).

The sum of verified and DCO flagged cases appropriately did not exceed 100% for any district, age group or site. There were no uniquely female cancers recorded in men or vice versa. In 530 cases (1.9%) recorded, date of registration preceded the date of diagnosis.

### DISCUSSION

While there is agreement about the value of quality assurance in cancer registries, clearly this must be standardized and universally

© Cancer Research Campaign 1997

**Table 6** Quality indicators in Mersey Regional Cancer Registry 1990–91: age group

| Age group (years) | Cases | Verification of diagnosis[a] | | DCO flag[b] | | Primary site unknown | | Diagnosis to registration interval (weeks) | | |
|---|---|---|---|---|---|---|---|---|---|---|
| | | % | (95% CL) | % | (95% CL) | % | (95% CL) | Median[c] | (95% CL) | 75th percentile |
| 0–4 | 50 | 74.0 | (61.2–86.2) | 10.0 | (1.7–18.3) | 2.0 | (0.0–5.9) | 27 | (17–30) | 40 |
| 5–9 | 20 | 80.0 | (62.5–97.5) | 5.0 | (0.0–14.6) | 0 | | 28 | (16–35) | 42 |
| 10–14 | 28 | 89.3 | (77.8–100) | 3.6 | (0.0–10.4) | 0 | | 25 | (17–34) | 42 |
| 15–19 | 90 | 93.3 | (88.2–98.5) | 1.1 | (0.0–3.3) | 0 | | 20 | (15–18) | 34 |
| 20–24 | 396 | 96.7 | (94.9–98.5) | 1.3 | (0.2–2.4) | 0.3 | (0.0–0.8) | 12 | (11–13) | 25 |
| 25–29 | 667 | 97.5 | (96.3–98.6) | 0.7 | (0.1–1.4) | 0 | | 13 | (12–13) | 23 |
| 30–34 | 839 | 97.5 | (96.4–98.6) | 1.4 | (0.6–2.2) | 0.2 | (0.0–0.6) | 13 | (12–13) | 23 |
| 35–39 | 709 | 94.5 | (92.8–96.2) | 1.0 | (0.3–1.7) | 0.4 | (0.0–0.9) | 12 | (11–13) | 20 |
| 40–44 | 874 | 94.2 | (92.6–95.7) | 1.8 | (1.0–2.7) | 0.3 | (0.0–0.7) | 13 | (12–13) | 23 |
| 45–49 | 1080 | 91.9 | (90.3–93.6) | 2.0 | (1.2–2.9) | 0.3 | (0.0–0.6) | 11 | (11–12) | 20 |
| 50–54 | 1571 | 87.7 | (86.1–89.3) | 3.9 | (3.0–4.9) | 1.2 | (0.7–1.8) | 10 | (9–10) | 17 |
| 55–59 | 2061 | 84.4 | (82.9–85.9) | 5.4 | (4.4–6.4) | 0.8 | (0.4–1.2) | 10 | (9–10) | 17 |
| 60–64 | 3195 | 82.0 | (80.7–83.3) | 6.4 | (5.5–7.2) | 1.2 | (0.8–1.6) | 10 | (9–10) | 16 |
| 65–69 | 3964 | 76.8 | (75.5–78.1) | 9.0 | (8.1–9.9) | 1.3 | (1.0–1.7) | 9 | (8–9) | 17 |
| 70–74 | 3793 | 73.0 | (71.6–74.4) | 10.2 | (9.2–11.1) | 1.3 | (1.0–1.7) | 9 | (8–9) | 16 |
| 75–79 | 3893 | 70.7 | (69.3–72.1) | 11.6 | (10.6–12.6) | 1.8 | (1.4–2.2) | 9 | (8–9) | 17 |
| 80–84 | 2786 | 64.1 | (62.3–65.9) | 16.2 | (14.8–17.6) | 2.9 | (2.3–3.5) | 8 | (7–8) | 15 |
| 85–89 | 1391 | 55.1 | (52.5–57.8) | 20.1 | (18.0–22.2) | 2.9 | (2.1–3.8) | 8 | (7–8) | 16 |
| 90 + | 522 | 45.2 | (40.9–49.5) | 23.0 | (19.4–26.6) | 4.2 | (2.5–5.9) | 7 | (6–8) | 15 |
| All ages | 27 942 | 77.3 | (76.8–77.8) | 8.9 | (8.6–9.3) | 1.5 | (1.3–1.6) | 10 | (10–10) | 17 |

[a]Verification includes pathological and cytological proof as well as proof by 'special test'. [b]The Registry DCO flag overestimates 'true' DCO registrations – see text. [c]Spearman correlation coefficient = 0.1375, $P < 0.0001$. 95% CL, 95% confidence limits.

**Table 7** Quality indicators in Mersey Regional Cancer Registry 1990–91: site

| Site (ICD-O) | Cases | Verification of diagnosis[a] | | DCO-flag[b] | | Diagnosis to registration interval (weeks) | | |
|---|---|---|---|---|---|---|---|---|
| | | % | (95% CL) | % | (95% CL) | Median[c] | (95% CL) | 75th percentile |
| Lip, mouth and pharynx (140–149) | 469 | 90.6 | (87.0–93.2) | 3.6 | (1.9–5.3) | 11 | (10–12) | 23 |
| Oesophagus (150) | 623 | 71.7 | (68.2–75.2) | 13.8 | (11.1–16.5) | 8 | (7–9) | 14 |
| Stomach (151) | 1217 | 70.9 | (68.3–73.5) | 13.1 | (11.2–15.0) | 8 | (7–8) | 14 |
| Colon (153) | 1790 | 75.1 | (73.1–77.1) | 11.4 | (9.9–12.9) | 9 | (8–9) | 16 |
| Rectum (154) | 1070 | 84.8 | (82.7–87.0) | 6.9 | (5.4–8.4) | 9 | (8–9) | 16 |
| Larynx (161) | 228 | 90.8 | (87.1–94.6) | 2.2 | (0.29–4.1) | 9 | (7–9) | 16 |
| Trachea, bronchus and lung (162) | 4398 | 55.2 | (53.7–56.7) | 15.6 | 14.6–16.7 | 8 | (7–8) | 15 |
| Haematopoietic (169) | 921 | 67.4 | (64.4–70.4) | 22.6 | (19.9–25.3) | 10 | (8–11) | 25 |
| Skin (non-melanoma) (173) | 4627 | 92.9 | (92.2–93.6) | 0.4 | (0.2–0.6) | 10 | (10–10) | 16 |
| Female breast (174) | 2749 | 85.2 | (83.9–86.5) | 5.3 | (4.5–6.1) | 11 | (10–11) | 18 |
| Cervix uteri (180 ) | 2783 | 96.9 | (96.3–97.5) | 0.9 | (0.5–1.2) | 13 | (12–13) | 24 |
| Body uterus (182) | 355 | 90.7 | (87.7–93.7) | 5.6 | (3.2–8.0) | 10 | (9–11) | 20 |
| Ovary (183) | 460 | 79.1 | (75.4–82.8) | 8.3 | (5.7–10.8) | 10 | (9–12) | 21 |
| Prostate (185) | 1122 | 83.6 | (81.4–85.8) | 7.0 | (5.5–8.4) | 10 | (10–11) | 17 |
| Testis (186) | 117 | 95.7 | (92.0–99.4) | 2.6 | (0.0–5.4) | 11 | (8–11) | 23 |
| Bladder (188) | 1160 | 90.0 | (88.3–91.7) | 4.9 | (3.7–6.2) | 10 | (9–10) | 16 |
| Brain (191) | 399 | 65.7 | (61.0–70.4) | 14.0 | (10.6–18.1) | 16 | (14–18) | 26 |
| Unknown primary (199) | 406 | 28.1 | (23.7–32.5) | 56.4 | (51.6–61.2) | 4 | (3–4) | 9 |
| Other sites (within 140–208) | 3048 | 63.6 | (61.5–64.9) | 12.7 | (12.1–13.3) | 9 | (8–9) | 30 |
| All cancers (140–208) | 27 942 | 77.3 | (76.8–77.8) | 8.9 | (8.6–9.3) | 10 | (10–10) | 17 |

[a]Verification includes pathological and cytological proof as well as proof by 'special test' (11% of haematopoietic and 2.5% of brain cancers had proof by 'special test' – for other sites such proofs were negligible). [b]The Registry DCO flag overestimates 'true' DCO registrations – see text. [c]Kruskal–Wallis one-way analysis of variance of ranks: $\chi^2 = 1111$ (17 d.f.), $P < 0.0001$. 95% CL, 95% confidence limits.

applied to allow meaningful comparisons, interpreted in the context of registration practice and sufficiently disaggregated to facilitate improvement. This first attempt to apply published measures comprehensively at the local level should be viewed as complementary to larger, infrequent, endeavours such as *Cancer Incidence in Five Continents* (Parkin and Muir 1992) in attempting to bring quality assurance into practice. Although the assessment of overall quality in MCCR was constrained by the lack of comparable data from elsewhere, nevertheless significant variations were exposed, demanding clear action and inviting further explanation. A local baseline has now been established against which to set standards, negotiate improvement and monitor practice.

*© Cancer Research Campaign 1997* 

### Ascertainment

Overall MCCR R/M ratios for lung cancer remain consistent (1.11:1 in 1990/1 compared with 1.12:1 and 1.11:1 for men and women, respectively, in 1989), showing little improvement. Comparative published data on district R/M ratios are few but shows similar variation (Centre for Cancer Epidemiology, 1992). Regional comparisons of R/M ratios although published annually (OPCS, 1994) are robust when deaths are expected to equal incidence and are difficult to interpret otherwise. For rapidly fatal cancers, R/M ratios under one imply underascertainment and values over one suggest duplication whereas, for other cancers, R/M ratios of 1:1 may signal underascertainment. The R/M ratio incorporates DC diagnostic uncertainty and for most sites lacks an explicit optimum. Furthermore, using the R/M ratio for rapidly fatal cancers to reflect overall ascertainment wrongly assumes that their ascertainment is typical.

There is little UK literature using capture–recapture to estimate cancer registry ascertainment. Ascertainment in MCCR 59% apparently compares unfavourably with results from Ontario 59–95% (Robles et al, 1988). However, Robles' study was reported 6 years after the incident year (compared with 8 months in our study), leading to more observed cases and more overlap between sources (e.g. death certificate and pathology), particularly affecting sites with longer survival such as breast and cervical cancer. Also, the accurate identification of source in Robles' study allowed three sources to be used (compared with two in our study), thereby strengthening the method. The method is most robust when capture and recapture give similarly sized groups, are clearly independent and have considerable overlap. Capture–recapture fails when there is a single source (e.g. for testicular cancer, which has excellent survival) and overestimates ascertainment when sources are mutually dependent (e.g. between death certificate and post-mortem data). Capture–recapture results here may thus illustrate the method's shortcomings as much as they measure local ascertainment. However, the method deserves further testing (perhaps against independent clinical case registers) and could be refined for cancer registry use by specifying the time interval after the incident year, using multiple sources, considering sites individually and adjusting for survival time.

Both R/M ratios and capture–recapture depend on multiple sources of information and, although probably less accurate, are easier to apply routinely than more resource intensive comparisons with clinical case registers (which tend to be site specific and have limited population coverage) or data reabstraction methods.

The DCD% estimated from audited registrations after death was preferred to the more familiar DCN%, which refers to cases *first* notified by DC. The DCD%, in identifying uniquely DC notified cases, is the better measure of ascertainment as 'accidents of timing', occurring when DC information apparently arrives in the Registry first, are eliminated. In MCCR, death certificates are processed before information from other sources to maximize the likelihood of successful traceback. Thus, rapidly fatal cancers in particular may be notified first, but not uniquely, by DC, and data processing backlogs, common to many registries, make ascertainment measured by DCN% appear worse by affecting timeliness. Overall DCD% and DCN% were estimated as 20.0% and 29.5%, respectively, illustrating the potential for distortion. Routine description of the DCD% is impossible, however, without information about notification source. There are no comparable data from other registries.

Approximately 10% of records selected for audit were 'not in file' mainly through removal for research and (paradoxically) verification purposes. Filing has since been reorganized!

### Completeness

The overall proportion with primary site unknown (1.5%) appears to have improved compared with levels of 6.23% and 6.95% for men and women respectively (Parkin and Muir, 1992), although the best district value of 0.8% indicates that further local improvement is possible. Improvement may reflect extensions in the range and application of diagnostic techniques. Although there are no comparable published reports about other missing key information, the overall proportions in MCCR appear acceptably low.

The DCO% measures completeness, as key data items such as date of diagnosis are missing, and validity (see on) mainly by affecting diagnostic accuracy. The effect of these deficiencies on incidence and survival analyses clearly depends upon the magnitude of the DCO% and the degree to which DCO cases are atypical. DCO registrations represent ascertainment failures comprising genuine diagnoses after death and cases unreported in life for which traceback has either been unsuccessful or has not been attempted. In MCCR, DCO registrations (7.4%) were overestimated by the DCO flag (8.9%). Misclassification arose through clerical failure to reset date of diagnosis (from date of death) when additional information was found, with clear implications for training. DC-independent cases, incorrectly DCO flagged, arose through data processing backlogs during recomputerization, which differentially affected sources other than death certificates. Similar mechanisms probably account for the variation in the positive predictive value of the DCO flag with death to registration interval. In MCCR, variations in the DCO-flagged proportion by site, age and district are likely to be a reasonable reflection of true DCO% variation (positive predictive value 83%) and raise issues for exploration with data providers.

Traceback was successful in 3533 out of 5601 (63.1%) DCD cases, thereby improving validity and completeness, as otherwise all DCD registrations would eventually be classified DCO. The DC-plus% estimated here provides a new marker of ante-mortem reporting failure. Less biased than DCO registrations, most DC-plus cases are randomly 'missed pathology', causing delays rather than failures in record completion, and may be indistinguishable in content from registrations completed chronologically. There are no comparable figures from other registries.

### Timeliness

MCCR registration timeliness compares favourably with elsewhere, with median diagnosis to registration intervals of 10 weeks and 24 weeks for MCCR and South Thames respectively (Thames Cancer Registry, 1995). In MCCR, statistically significant variations in the diagnosis to registration interval are highlighted by site, district and age group. Explanations for delays include complex childhood cancer records not being available to registration clerks, routine failure to notify histological diagnoses, especially in district 7, and a greater proportion of 'faster' DC notifications among older people and in some districts. The implications of these findings need to be explored with individual providers if registration is to be improved. Commonly registered more promptly that registrations via pathology, DCO cases should

© Cancer Research Campaign 1997

ideally be excluded from calculations of timeliness, but they were known only for the audited records, precluding their exclusion here. Comparisons with other registries were limited by the lack of published data, although South Thames data also showed some variation with site and district (Thames Cancer Registry, 1995).

The diagnosis to registration interval (expressed as median and 75th percentile) uses routinely available data from the Cancer Minimum Data Set (NHS Management Executive, 1992) and depends upon ascertainment and the elapsed time from the incident year. This time could be standardized to allow more meaningful comparison between registries, thereby better reflecting the process of registration.

### Validity

Variations in the proportion of histologically verified diagnoses by site (Centre for Cancer Epidemiology, 1992; Parkin and Muir, 1992) and age (Parkin and Muir, 1992) are confirmed, and district variations are reported that illuminate cause. The histologically verified proportion assesses the validity of registration data (Centre for Cancer Epidemiology, 1992; Parkin and Muir, 1992; Black et al, 1993) but again lacks clear definition, hampering comparisons. The inclusion or exclusion of haematology and cytology reports alters the proportion of records defined as 'histologically' verified. This problems applies especially to haematopoietic and cervical cancers, in which treatment decisions are routinely based on blood and cytology reports. This may partly explain our finding of apparently lower verification in children and young adults. An alteration of terminology is suggested. Instead of using the term 'histological verification', verification of diagnosis could be given three broad levels: microscopic, specific biochemical and imaging techniques; and clinical. Each level requires exclusive definition to include all diagnostic techniques and should keep pace with technological change.

The DCO% as a validity measure is complementary to verification of diagnosis (Parkin and Muir, 1992). Previously reported inconsistencies in the DCO complicate the interpretation of international comparisons of incidence (Parkin et al, 1994) and survival (Berrino et al, 1995). Differences between UK regions probably also result from variable traceback procedures. The significant district variation found in MCCR is, however, likely to be attributable to differences in other factors, such as demography, incidence, clinical management and survival between population subgroups and clearly needs further work.

International DCO% are published in 'Cancer Incidence in Five Continents' (Parkin and Muir, 1992), although they were omitted for MCCR recently (1983–87 data) precluding accurate comparison. DCO% appear in some, but not all, registry reports and are generally lower than in MCCR but show similar variation by site (Black et al, 1993; Thames Cancer Registry, 1995), district (Centre for Cancer Epidemiology, 1992) and age (Parkin and Muir, 1992). The shorter time interval (8 months) for successful traceback between the end of the study period and the creation of the study dataset partly explains the high overall DCO%, contrasting with intervals of over 3 years elsewhere (Centre for Cancer Epidemiology, 1992; Parkin and Muir, 1992; Black et al, 1993; Thames Cancer Registry, 1995).

Inconsistencies in the DCO% should be resolved by stricter definition. Conceptually, the DCO is a registration in which 'no information other than the death certificate is available' (Jensen et al, 1991). Availability is relative and time dependent – whether additional information can be found varies with registry effort and resources. Pragmatically, the definition 'DCO' could apply to a record containing only DC information, for example 24 months after death, making the DCO% the proportion of records in which 'only death certificate information is held at 24 months after death, despite specified search by the registry'.

Improbable or impossible dates are rarely revealed in registry reports. In MCCR, 2% of records were apparently registered before their diagnosis. Local discussion suggests that this arises when dates are imputed by computer software when information is missing, and through keyboard errors.

### CONCLUSION

Although the importance of high-quality cancer registration has been highlighted (Day and Davies, 1996), the means of achievement seems less clear. Accurate cancer registry information is essential for the definition, development and monitoring of Cancer Units and Centres (Department of Health, 1994), and quality now forms part of the national core contract (NHS Executive, 1996) for cancer registries. While attempting to put quality assurance into practice at local level, this study has demonstrated shortcomings in the scope, definition and use of existing quality assurance measures, and the absence of explicit standards. Suggestions are made about clarifying terminology, standardizing existing definitions and adding new measures. Without this, national and international data comparisons are impoverished, variations can be ignored as artefactual and data do not improve, ultimately casting doubt on their use. The measures used here could, with further development, form the basis of national quality assurance, alongside the much needed standardization of registry procedures. Custom and practice must now give way to a systematic and standardized approach to quality assurance in cancer registries.

### ACKNOWLEDGEMENTS

We would like to acknowledge the staff of the Merseyside and Cheshire Cancer Registry and all those who provide data to the Registry.

### REFERENCES

Balarajan R and Scott A (1983) National cancer registration: an appraisal. *Community Med* **6**: 31–37

Benn RT, Leck I and Nwene UP (1982) Estimation of completeness of cancer registration. *Int J Epidemiol* **11**: 362–367

Berrino F, Estève and Coleman MP (1995) Basic issues in estimating and comparing the survival of cancer patients. In *Survival of Cancer Patients in Europe: The EUROCARE study*. Berrino F, Sant M, Verdecchia A, Capocaccia R, Hakulinen T and Estève J (eds), pp. 1–14. IARC Scientific Publications: Lyon

Black RJ, Sharp L and Kendrick SW (1993) *Trends in Cancer Survival in Scotland 1968–1990*. Information & Statistics Division, Directorate of Information Services, National Health Service in Scotland: Edinburgh

Centre for Cancer Epidemiology (1992) *Cancer in the North West*. Centre for Cancer Epidemiology: Manchester

Day NE and Davies TW (1996) Cancer Registration: integrate or disintergrate (editorial). *Br Med J* **313**: 896

Department of Health (1994) *A Policy Framework for Commissioning Cancer Services*. A consultative document from the expert advisory group on cancer. May 1994.

Freedman LS (1978) Variations in the level of reporting by hospitals to a regional cancer registry. *Br J Cancer* **37**: 861–865

Gardner MJ and Altman DG (1992) *Statistics with Confidence*. BMJ: London

Goldberg J, Gelfand MG and Levy PS (1980) Registry evaluation methods: a review and case study. *Epidem Rev* **56**: 357–359

© Cancer Research Campaign 1997

Hawkins MM and Swerdlow AJ (1992) Completeness of cancer and death follow-up obtained through the National Health Service Central Register for England and Wales. *Br J Cancer* **66**: 408–413

Jensen OM, Parkin DM, MacLennan R, Muir CS and Skeet RG (1991) *Cancer Registration: Principles and Methods.* IARC Scientific Publication: Lyon

Mcleod WT and Hanks P (eds) (1982) Collins Concise English Dictionary. Collins: Glasgow

NHS Management Executive (1992) *Minimum Data Set for the National Cancer Registration System.* Letter EL(92)95, 18 December 1992. HMSO: London

NHS Executive (1996) *Core Contract for Purchasing Cancer Registration.* Letter EL(96)7, 9 February 1996. HMSO: London

Office of Population Censuses and Surveys (1990) *Review of the National Cancer Registration System.* Series MB1 No. 17. HMSO: London

Office of Population Censuses and Surveys (1994) *Cancer Statistics.* Series MB1 No. 22. HMSO: London

Parkin DM and Muir CS (1992) Comparability and quality of data. In *Cancer Incidence in Five Continents* Vol. 6, Parkin DM, Muir CS, Whelan SL, Gao YT, Ferlay J and Powell J. (eds), pp. 45–56. IARC Scientific Publication: Lyon

Parkin DM, Chen VW, Ferlay J, Galceran J, Storm HH and Whelan SL (1994) *Comparability and quality control in Cancer Registration.* IARC Technical Report No. 19: Lyon, France

Robles SC, Marrett LD, Clarke EA and Risch HA (1988) An application of capture recapture methods to the estimation of completeness of cancer registration. *J Clin Epidemiol* **41**: 495–501

Skeet RG (1991) Quality and quality control. In *Cancer Registration: Principles and Methods*, Jensen OM, Parkin DM, MacLennan R, Muir CS and Skeet RG. (eds), pp. 101–107. IARC Scientific Publication: Lyon

Swerdlow AJ (1986) Cancer registration in E&W: some aspects relevant to the interpretation of data. *J R Stat Soc (Series A)* **149**: 146–160

Thames Cancer Registry (1992) *Cancer in North West Thames 1987–9. Cancer in North East Thames 1987–9. Cancer in South West Thames 1987–9. Cancer in South East Thames 1987–9.* Thames Cancer Registry: Sutton, UK

Thames Cancer Registry (1995) *Cancer in South East England 1992.* Thames Cancer Registry: Sutton UK

Youngson JH, Ashby D and Williams EMI (1992) *Incidence of Cancer in Mersey Region and its Constituent Health Districts, 1986–90.* Mersey Regional Cancer Registry: Liverpool

### APPENDIX: TWO SOURCE CAPTURE–RECAPTURE ESTIMATION

### 1. Robles' two-source calculation

$n_1$ = number ascertained by source 1
$n_2$ = number ascertained by source 2
$t$ = total ascertained from sources 1 and 2 combined
$c$ = overlap $(n_1 + n_2 - t)$
$N$ = estimated total cases using capture–recapture principles = $\frac{(n_1 \times n_2)}{c}$

$t/N$ = estimated ascertainment

Variance of $N = \text{var}_N = \frac{n_1 \times n_2 (t - n_1)(t - n_2)}{c^3}$

Using s.e. = $\sqrt{\text{var}}$, the 95% confidence limits for the estimate of ascertainment are:

$$\left(\frac{t}{N + 1.96\sqrt{\text{var}_N}}\right) \text{ and } \left(\frac{t}{N - 1.96\sqrt{\text{var}_N}}\right)$$

Example: All sites, Mersey Regional Cancer Registry (MRCR), 1990–91:

$n_1 = 16379$ (source pathology) $n_2 = 12734$ (source death)

$t = 24681$ $c = 4432$

Therefore $N = \frac{16379 \times 12734}{4432} = 47060$ and $\frac{t}{N} = \frac{24681}{47060} = 52.4\%$

$$\text{var}_N = \frac{16379 \times 12734(24681 - 16379)(24681 - 12734)}{4432^3} = 237626$$

Confidence limits are

$$\left(\frac{24681}{47060 + 1.96\sqrt{237626}}\right) \text{ and } \left(\frac{24681}{47060 - 1.96\sqrt{237626}}\right)$$

Estimated ascertainment is thus 52.45% (51.4–53.5%).

### 2. Modified two-source estimation used for MRCR

Use $T$ = total cases ascertained from all sources available to registry and calculate estimated ascertainment as $\frac{T}{N}$

Now $T = 27\,942$ for all cases in MRCR 1990–91

Therefore, estimated ascertainment for all sites = $\frac{27942}{47060} = 59.4\%$

And its confidence limits are

$$\left(\frac{27942}{47060 + 1.96\sqrt{237626}}\right) \text{ and } \left(\frac{27942}{47060 - 1.96\sqrt{237626}}\right)$$

Estimated ascertainment is thus 59.4% (57.2–60.6%).

 © Cancer Research Campaign 1997


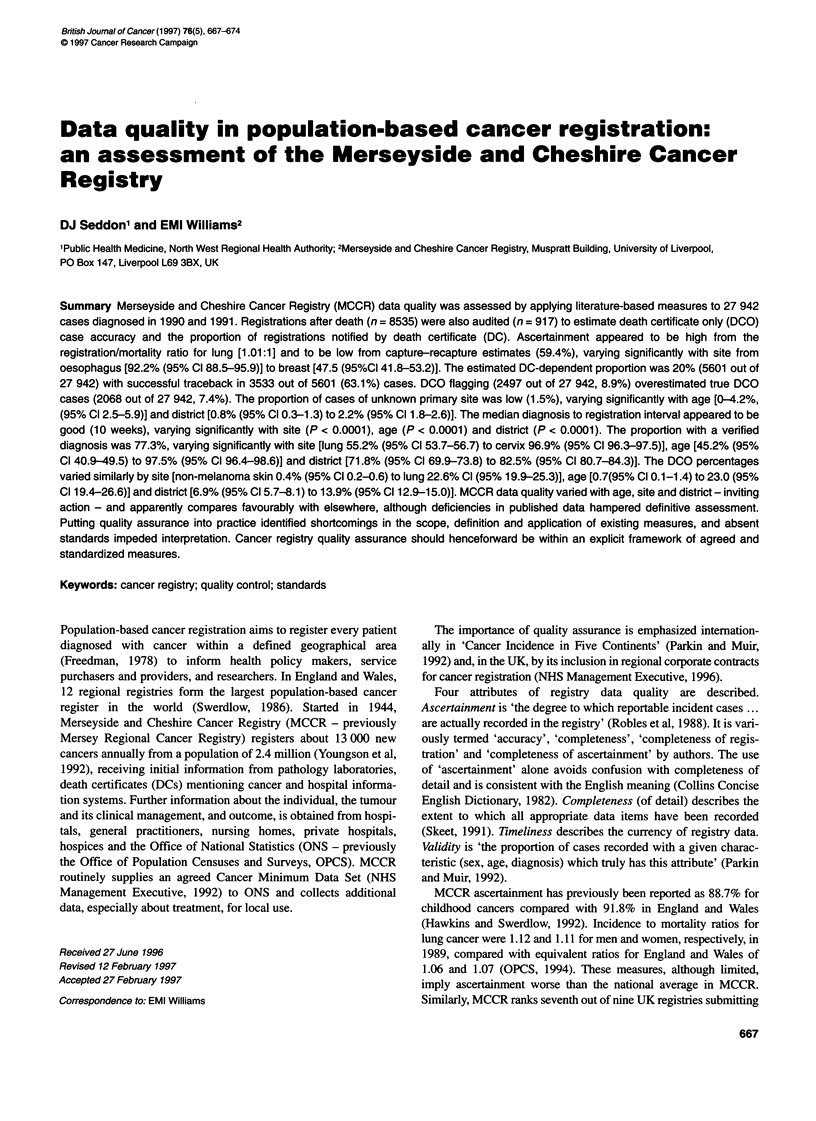

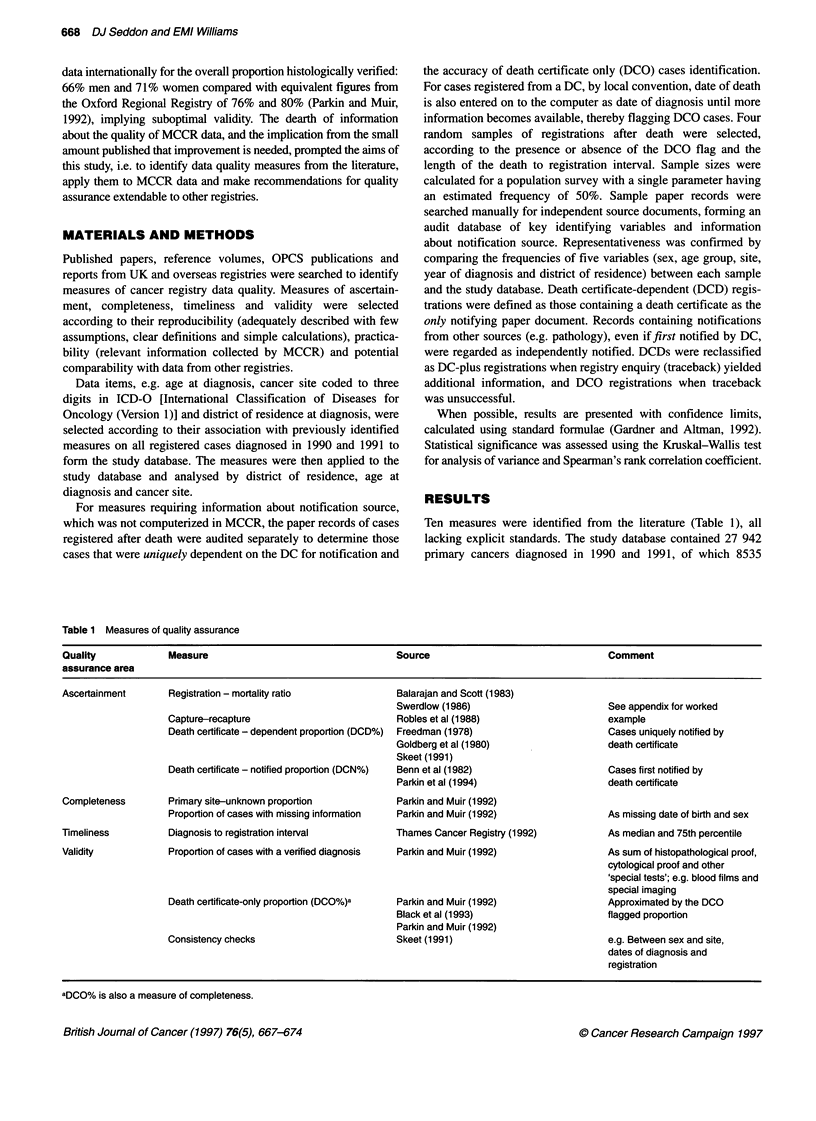

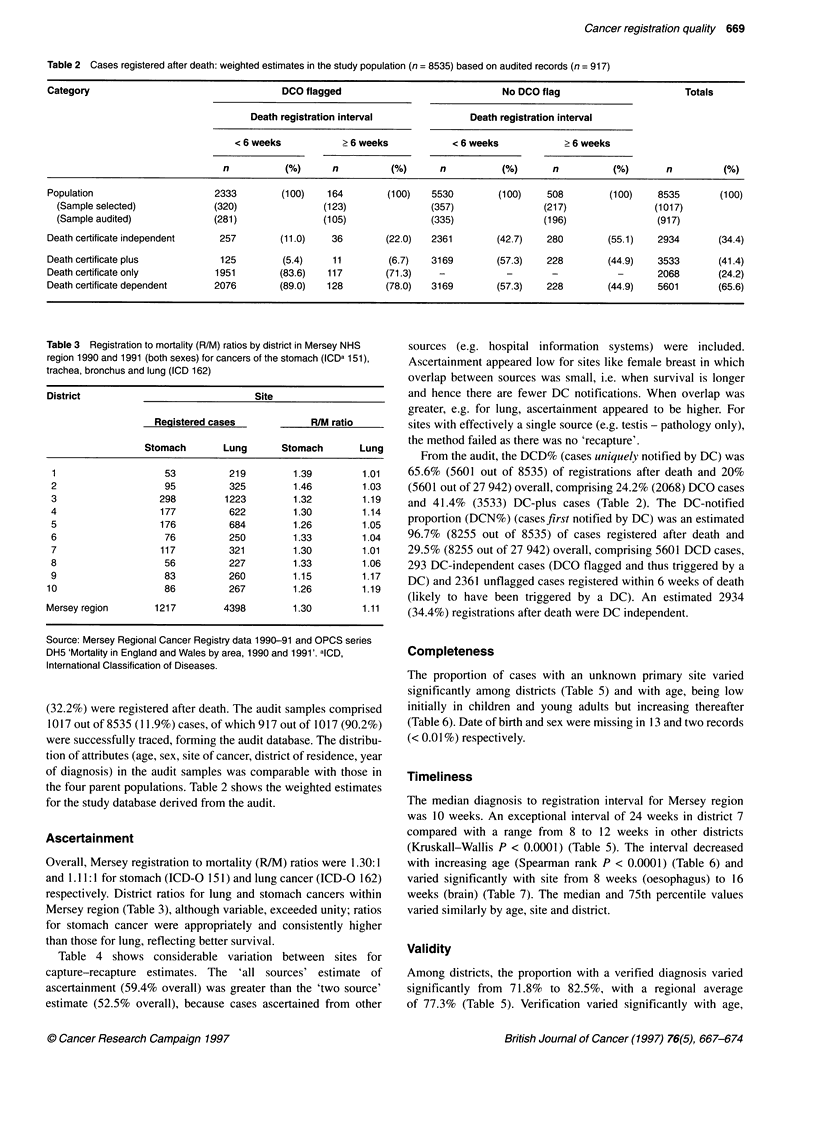

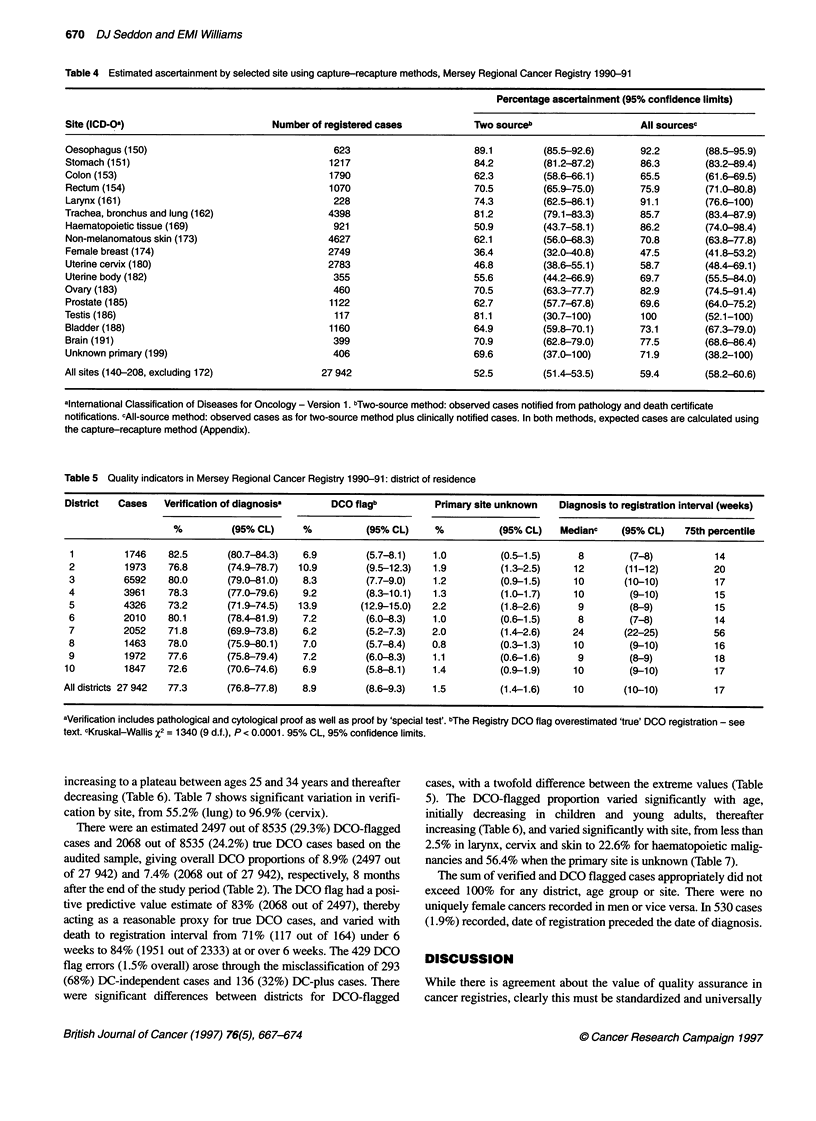

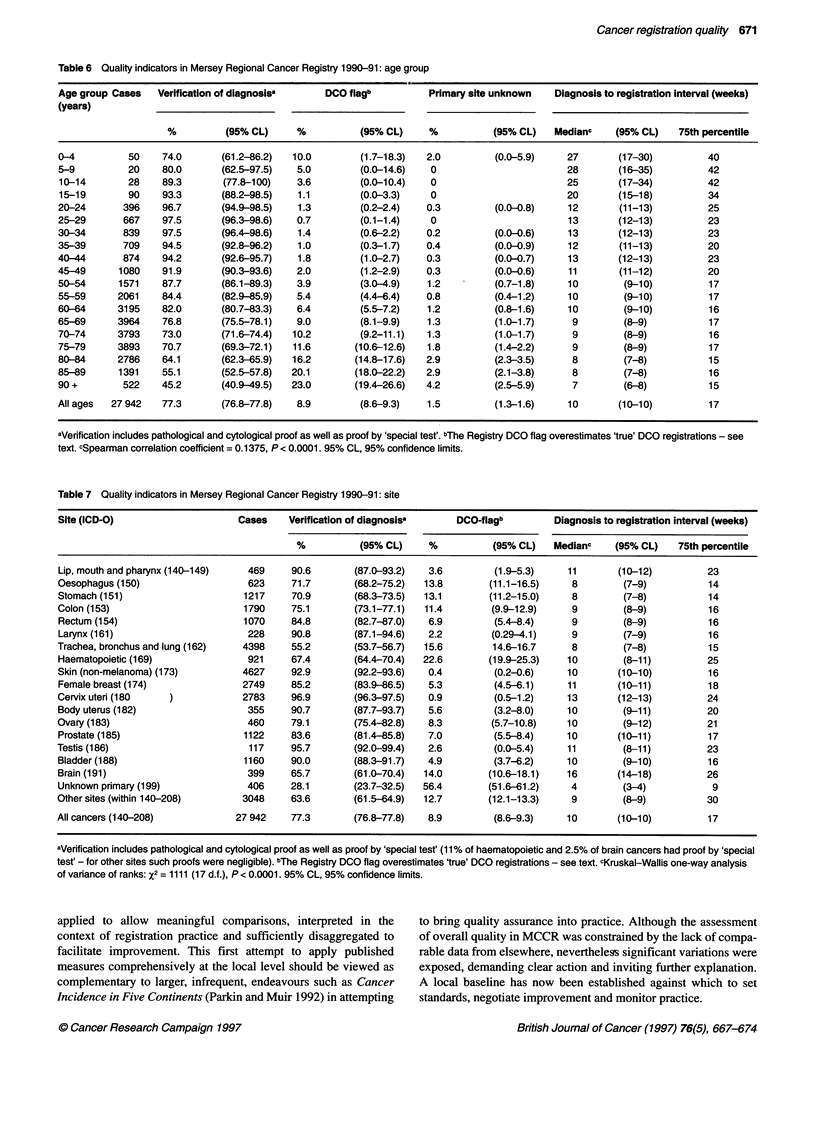

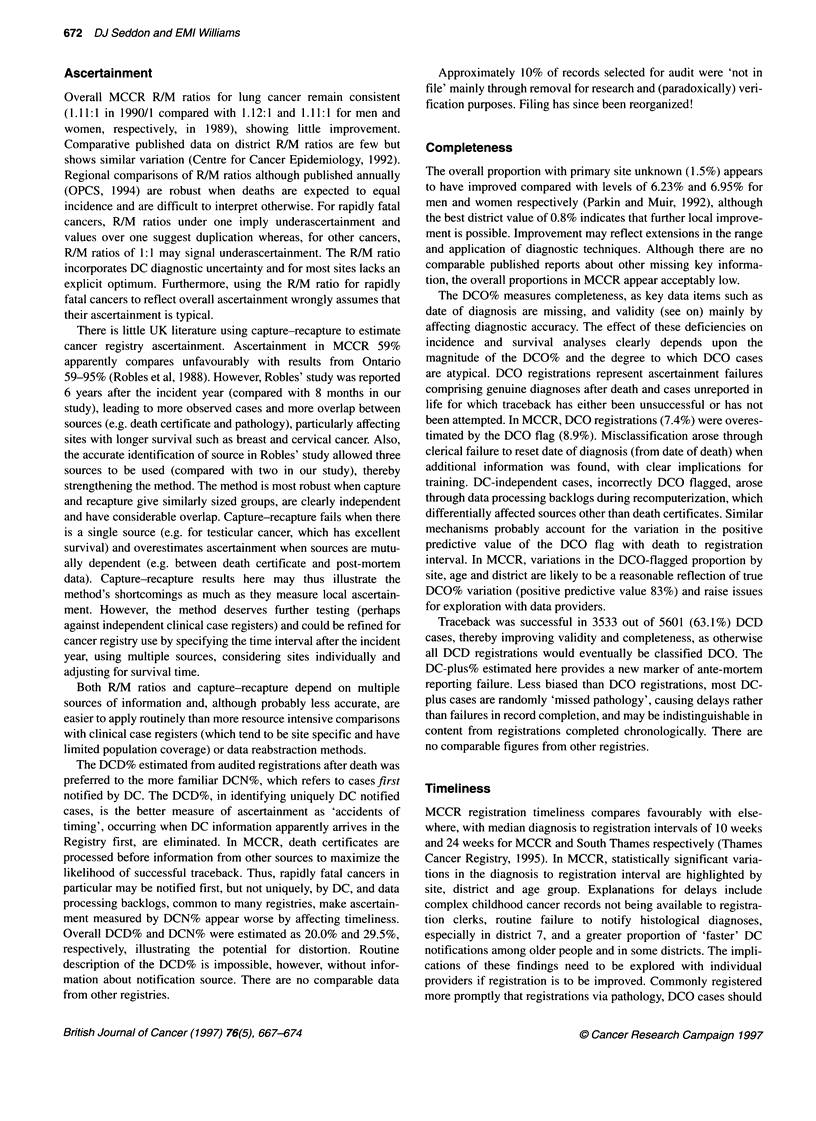

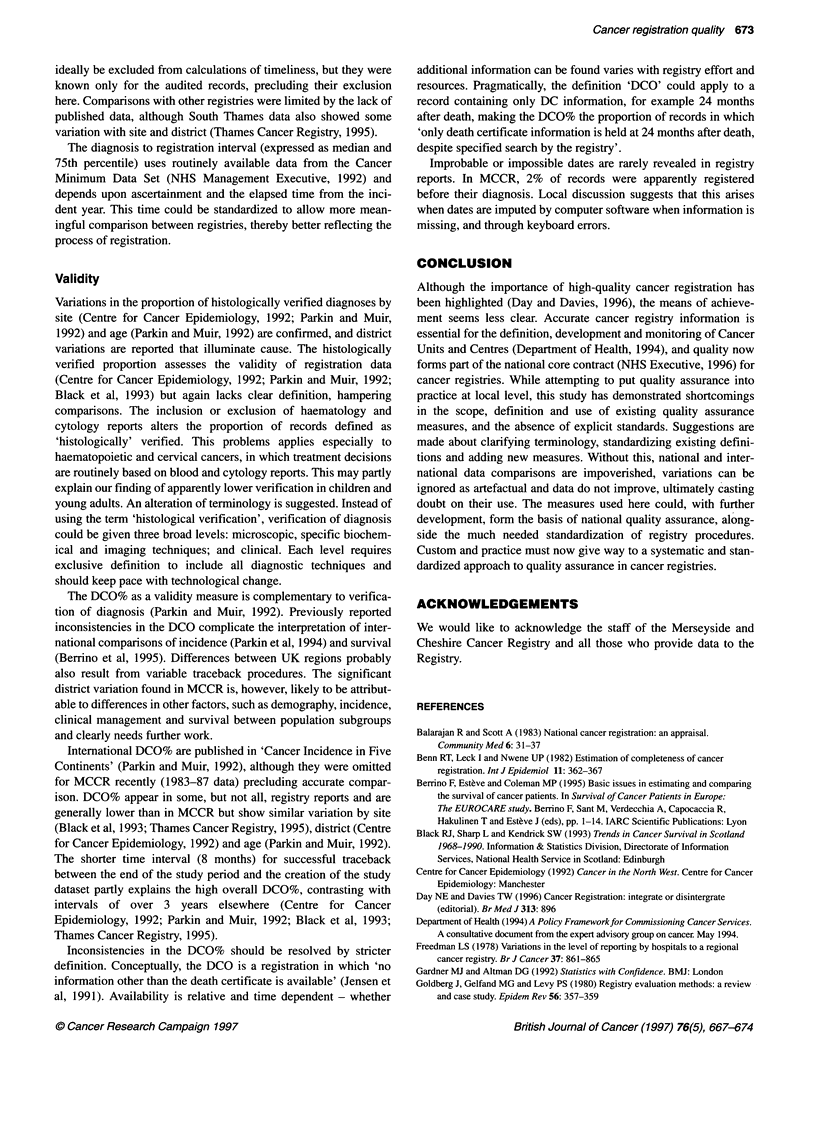

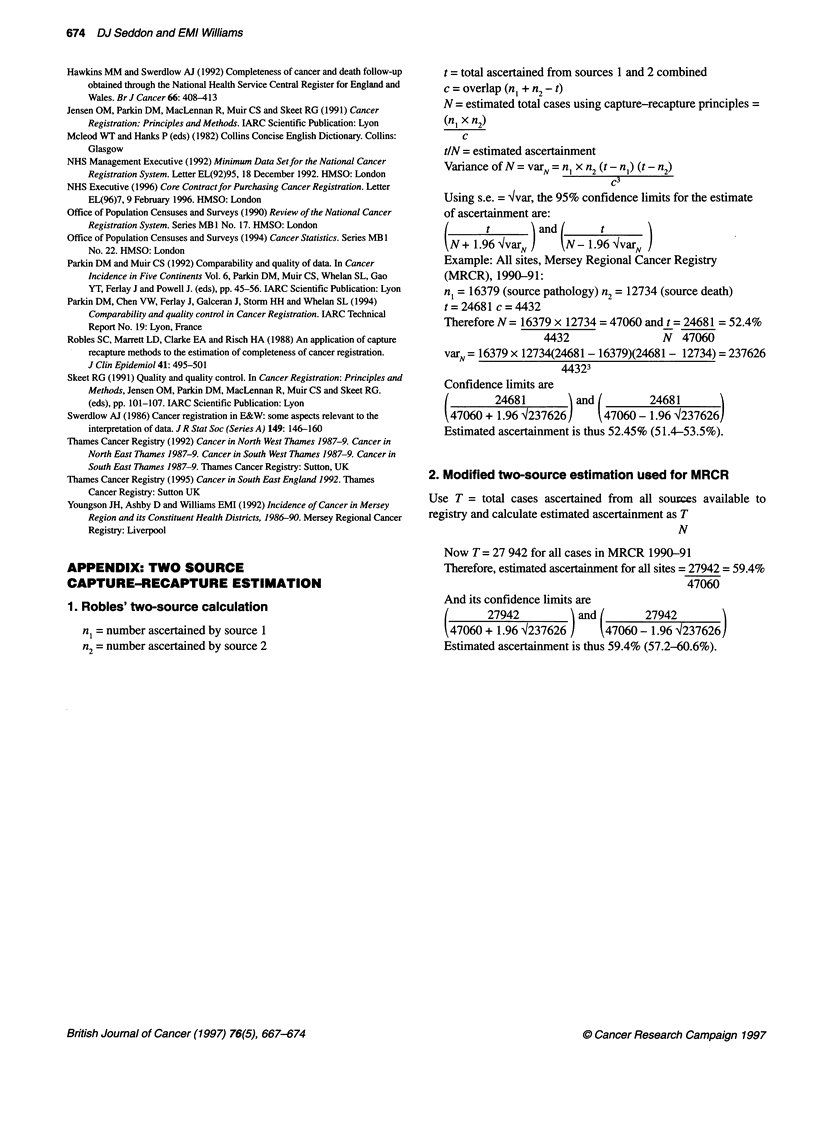


## References

[OCR_00735] Balarajan R., Scott A. (1983). National cancer registration: an appraisal.. Community Med.

[OCR_00739] Benn R. T., Leck I., Nwene U. P. (1982). Estimation of completeness of cancer registration.. Int J Epidemiol.

[OCR_00758] Day N. E., Davies T. W. (1996). Cancer registration: integrate or disintegrate?. BMJ.

[OCR_00765] Freedman L. S. (1978). Variations in the level of reporting by hospitals to a regional cancer registry.. Br J Cancer.

[OCR_00779] Hawkins M. M., Swerdlow A. J. (1992). Completeness of cancer and death follow-up obtained through the National Health Service Central Register for England and Wales.. Br J Cancer.

[OCR_00817] Robles S. C., Marrett L. D., Clarke E. A., Risch H. A. (1988). An application of capture-recapture methods to the estimation of completeness of cancer registration.. J Clin Epidemiol.

